# A Hybrid Decision Tree and Deep Learning Approach Combining Medical Imaging and Electronic Medical Records to Predict Intubation Among Hospitalized Patients With COVID-19: Algorithm Development and Validation

**DOI:** 10.2196/46905

**Published:** 2023-10-26

**Authors:** Kim-Anh-Nhi Nguyen, Pranai Tandon, Sahar Ghanavati, Satya Narayana Cheetirala, Prem Timsina, Robert Freeman, David Reich, Matthew A Levin, Madhu Mazumdar, Zahi A Fayad, Arash Kia

**Affiliations:** 1 Institute for Healthcare Delivery Science Icahn School of Medicine at Mount Sinai New York, NY United States; 2 Department of Medicine Division of Pulmonary, Critical Care, and Sleep Medicine Icahn School of Medicine at Mount Sinai New York, NY United States; 3 Hospital Administration Icahn School of Medicine at Mount Sinai New York, NY United States; 4 Department of Anesthesiology, Perioperative and Pain Medicine Icahn School of Medicine at Mount Sinai New York, NY United States; 5 Department of Genetics and Genomic Sciences Icahn School of Medicine at Mount Sinai New York, NY United States; 6 Windreich Department of Artificial Intelligence and Human Health Icahn School of Medicine at Mount Sinai New York, NY United States; 7 Department of Population Health Science and Policy Icahn School of Medicine at Mount Sinai New York, NY United States; 8 BioMedical Engineering and Imaging Institute Icahn School of Medicine at Mount Sinai New York, NY United States; 9 Department of Radiology Icahn School of Medicine at Mount Sinai New York, NY United States

**Keywords:** COVID-19, medical imaging, machine learning, chest radiograph, mechanical ventilation, electronic health records, intubation, decision trees, hybrid model, clinical informatics

## Abstract

**Background:**

Early prediction of the need for invasive mechanical ventilation (IMV) in patients hospitalized with COVID-19 symptoms can help in the allocation of resources appropriately and improve patient outcomes by appropriately monitoring and treating patients at the greatest risk of respiratory failure. To help with the complexity of deciding whether a patient needs IMV, machine learning algorithms may help bring more prognostic value in a timely and systematic manner. Chest radiographs (CXRs) and electronic medical records (EMRs), typically obtained early in patients admitted with COVID-19, are the keys to deciding whether they need IMV.

**Objective:**

We aimed to evaluate the use of a machine learning model to predict the need for intubation within 24 hours by using a combination of CXR and EMR data in an end-to-end automated pipeline. We included historical data from 2481 hospitalizations at The Mount Sinai Hospital in New York City.

**Methods:**

CXRs were first resized, rescaled, and normalized. Then lungs were segmented from the CXRs by using a U-Net algorithm. After splitting them into a training and a test set, the training set images were augmented. The augmented images were used to train an image classifier to predict the probability of intubation with a prediction window of 24 hours by retraining a pretrained DenseNet model by using transfer learning, 10-fold cross-validation, and grid search. Then, in the final fusion model, we trained a random forest algorithm via 10-fold cross-validation by combining the probability score from the image classifier with 41 longitudinal variables in the EMR. Variables in the EMR included clinical and laboratory data routinely collected in the inpatient setting. The final fusion model gave a prediction likelihood for the need of intubation within 24 hours as well.

**Results:**

At a prediction probability threshold of 0.5, the fusion model provided 78.9% (95% CI 59%-96%) sensitivity, 83% (95% CI 76%-89%) specificity, 0.509 (95% CI 0.34-0.67) *F*_1_-score, 0.874 (95% CI 0.80-0.94) area under the receiver operating characteristic curve (AUROC), and 0.497 (95% CI 0.32-0.65) area under the precision recall curve (AUPRC) on the holdout set. Compared to the image classifier alone, which had an AUROC of 0.577 (95% CI 0.44-0.73) and an AUPRC of 0.206 (95% CI 0.08-0.38), the fusion model showed significant improvement (*P*<.001). The most important predictor variables were respiratory rate, C-reactive protein, oxygen saturation, and lactate dehydrogenase. The imaging probability score ranked 15th in overall feature importance.

**Conclusions:**

We show that, when linked with EMR data, an automated deep learning image classifier improved performance in identifying hospitalized patients with severe COVID-19 at risk for intubation. With additional prospective and external validation, such a model may assist risk assessment and optimize clinical decision-making in choosing the best care plan during the critical stages of COVID-19.

## Introduction

Severe COVID-19 caused by SARS-CoV-2 predominantly affects the lungs due to the high affinity of the virus for the angiotensin-converting enzyme 2 receptor expressed extensively in the alveolar epithelium [1]. Approximately 14% of patients with COVID-19 required hospitalization during the initial wave of the pandemic, and the intensive care unit transfer rate ranged from 5% to 32% [2,3]. Acute hypoxemic respiratory failure, complicated by acute respiratory distress syndrome, is a frequent cause of mortality among hospitalized patients with severe COVID-19. Thus, airway and ventilation management is crucial for optimizing patient outcomes [4]. There are several guidelines for the respiratory management of SARS-CoV-2 infection, supporting the emerging consensus that noninvasive ventilation and high-flow nasal cannula are superior to invasive mechanical ventilation (IMV) for treating COVID-19 acute hypoxemic respiratory failure [5-7]. IMV, however, may ultimately be required in 8%-20% of those hospitalized with COVID-19 [8-10].

The decision to intubate a patient with COVID-19 and the timings of intubation are very challenging, and there remains significant clinical uncertainty. Currently, clinical judgment, patient’s choice, and advance directives regarding IMV are the main drivers of the decision to intubate. Clinical markers such as respiratory rate, oxygen saturation, dyspnea, arterial blood gases, and radiographic observations are the primary markers routinely being used to identify candidates for intubation [11]. There is no traditionally agreed upon numeric score or index, and while certain indices have been proposed, such as the ratio of oxygen saturation index, their use is limited to certain samples, and these indices are in the early phase of clinical validation and adoption [12]. As such, opportunity exists for multimodal artificial intelligence methods to fill this gap.

**Figure 1:**
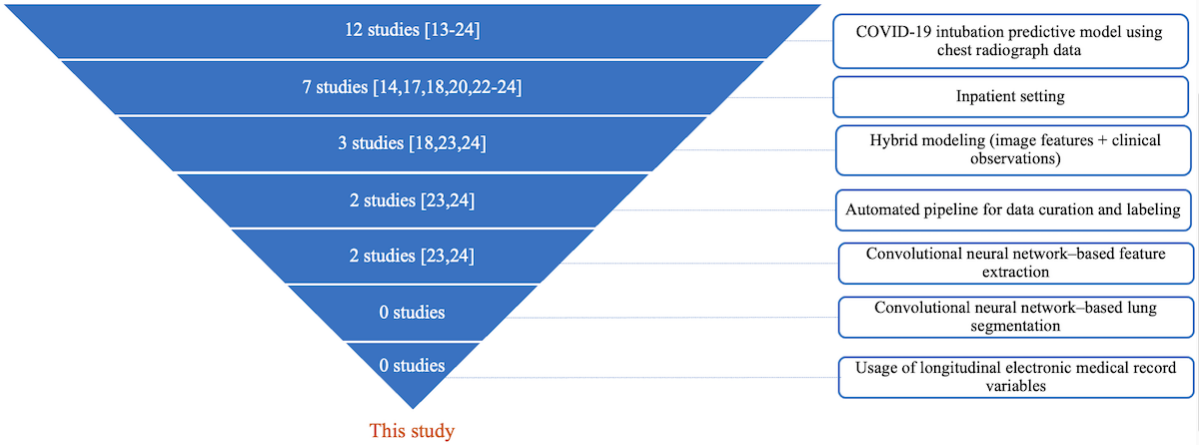
Funnel graph showing the number of similar published studies [13-24] by criteria of review.

Since 2020, many published studies [13-24] have tried to use machine learning techniques to predict the need for mechanical ventilation in patients with COVID-19. The majority of these studies used only clinical variables (structured data) [13] and only 15 of them (Figure 1 [13-24]) considered chest radiographs (CXRs) as a potential modality combined with clinical variables. Figure 1 is a funnel graph showing the number of similar published studies by criteria of review. The scope and top criteria for this study are “COVID-19 intubation predictive model using CXR data.” All referenced studies were found through the following PubMed query between January 1, 2020, and February 28, 2023: (“COVID-19” OR “coronavirus disease 2019”) AND (“artificial intelligence” OR “machine learning” OR “deep learning” OR “convolutional neural network”) AND (“chest x-ray” OR “chest radiograph”) AND (“intubation” or “mechanical ventilation”). Out of the 18 studies found, 6 were out of our study’s scope (different clinical outcome prediction or review type of studies). Each study was evaluated against each criterion. No study satisfied all the criteria except our study. Our new approach not only combined CXR data and clinical variables to predict the need for mechanical ventilation but also tried to show that applying automated image segmentation and using longitudinal values of clinical observations builds the prognostic potential in patients’ clinical profiles.

This study evaluates a machine learning risk stratification approach to predict the need for invasive ventilation based on a broad range of potential predictors. We designed a multimodality machine learning classifier based on electronic medical record (EMR) data and picture archiving and communication system images to predict the likelihood of intubation for patients with COVID-19 on the floor up to 24 hours in advance.

## Methods

### Study Population and Setting

**Figure 2:**
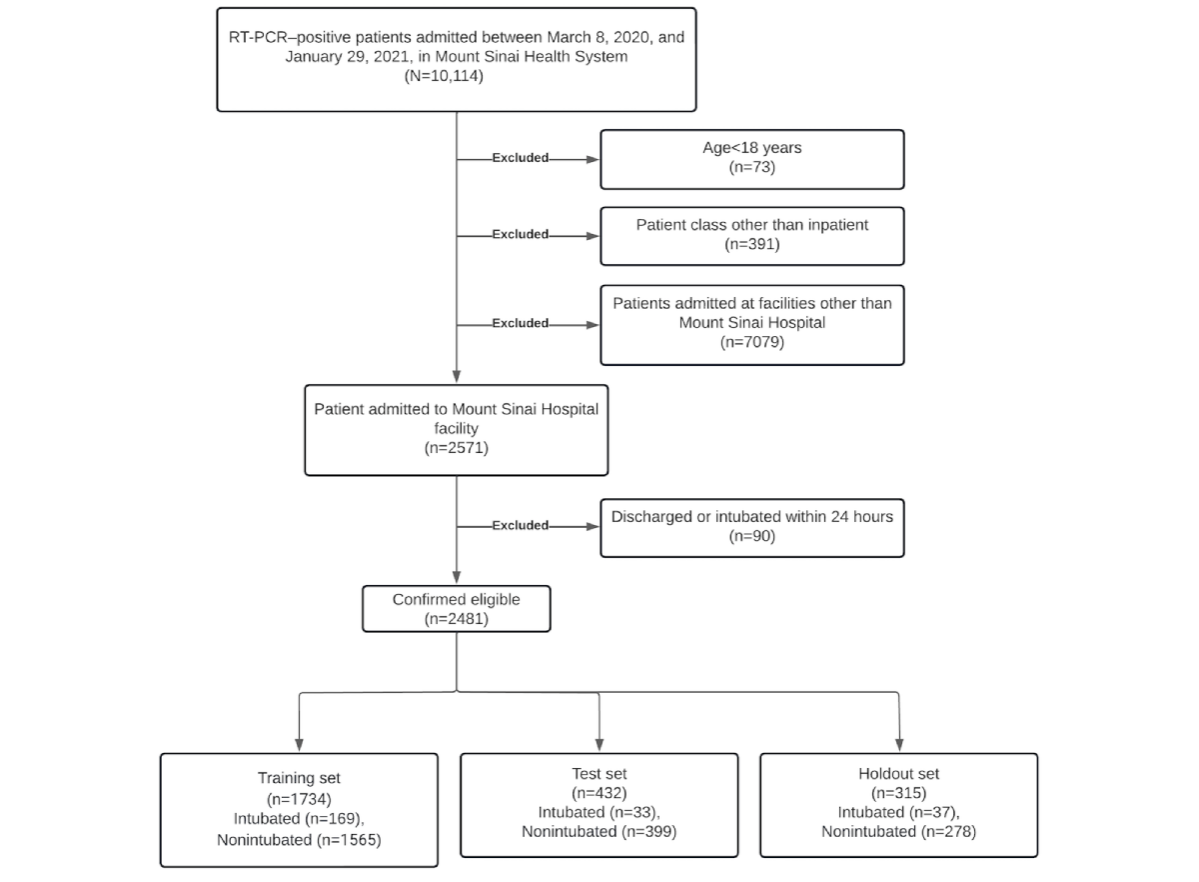
Inclusion and exclusion criteria chart. RT-PCR: reverse transcription polymerase chain reaction.

We included all adult patients (≥18 years of age) admitted to The Mount Sinai Hospital (New York, NY) between March 8, 2020, and January 29, 2021, with a confirmed COVID-19 diagnosis by real-time reverse transcription polymerase chain reaction at the time of admission. Patients who were intubated or discharged within 24 hours of admission were excluded. Figure 2 shows the flowchart of the inclusion and exclusion of the patients in this cohort. This study adhered to the TRIPOD (Transparent Reporting of a Multivariable Prediction Model for Individual Prognosis Or Diagnosis) statement [25].

### Ethics Approval

This study was undertaken at The Mount Sinai Hospital, a 1134-bed tertiary care teaching facility, and it was approved by the institutional research board (approval IRB-18-00581). All methods were performed in accordance with the relevant guidelines and regulations provided by the institutional research board, which granted a waiver of informed consent.

### Data Sources

The Mount Sinai Hospital currently uses 3 main electronic health record platforms: Epic (Epic Systems), Cerner (Cerner Corporation), and Laboratory Information Systems Suite (Single Copy Cluster Soft Computer). Data are aggregated from all 3 systems into a harmonized data warehouse. We received admission-discharge-transfer events from Cerner, laboratory results from Laboratory Information Systems Suite, and clinical data (ie, vital signs and nursing assessments) from Epic. Electrocardiogram results were obtained from the MUSE cardiology information system (GE HealthCare Technologies, Inc). To assemble the CXR data set, we obtained raw DICOM (Digital Imaging and Communications in Medicine) files from the picture archiving and communication system platform (GE HealthCare Technologies, Inc). CXRs taken in supine and upright positions were included.

### Label and Clinical Profile 

All inpatient encounters were annotated based on the following logic.

1. If the intubation happened within the inpatient hospital length of stay, the label was positive, and the label time stamp was the intubation time.
2. Otherwise, we consider that the patient was not intubated, and therefore, the label was negative, and the label time stamp was the discharge time.

Clinical profiles, including vital signs, laboratory results, nursing assessments, and electrocardiograms, were censored 24 hours before the label time stamp. The prediction window of 24 hours was chosen to provide a timely opportunity for clinical interventions, goals of care discussions, and resource planning.

### CXR Processing

We included radiograph images with computed radiography and digital radiography modalities in anteroposterior or posteroanterior views. For each patient with a CXR, we used the last segmented CXR before the prediction time stamp. The images were resized to 224×224 pixels; then, their pixel intensities were rescaled to range between 0 and 255, and histogram matching and normalization were performed on the intensities. To make sure that the deep learning model did not overfit and was robust, we applied oversampling of the minority class by augmenting each image in the training set by using a random combination of right or left rotation (maximum 15°), random flipping, random translation, random blurring, and random sharpening. The region of interest in the acquired CXR was the lungs (left and right). However, they were taken with some noise surrounding the lungs, including annotated text in the corners; external devices placed on the patient; and adjacent anatomy, including the shoulders, neck, and heart. We performed image segmentation in order to retain only the lung regions of the images.

**Figure 3:**
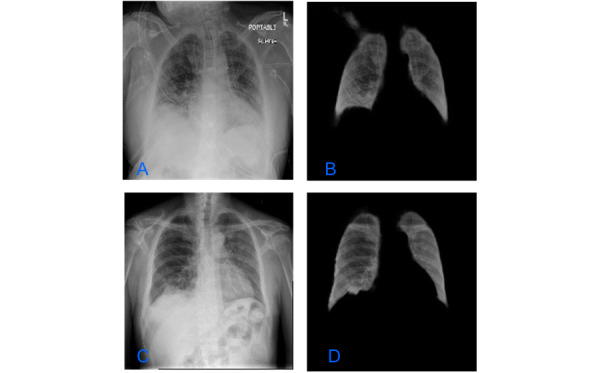
Chest radiographic images before (A and C) and after (B and D) image segmentation.

The CXRs were segmented using the U-Net model architecture [26], a fully connected convolutional neural network consisting of an encoder and a decoder. Specifically, we used the LungVAE [27] implementation of U-Net, which was trained on a publicly available CXR data set. Figure 3 shows a CXR before and after segmentation.

### Modeling and Localization Framework

#### Training, Testing, and Holdout Split

We randomly split the cohort into a training set (1734/2481, 70%), a test set (432/2481, 17%), and a holdout set (315/2481, 13%), with no patient overlap between the sets. Because the intubation rate was 9.6% (239/2481) over the whole cohort, there was an extreme class imbalance between the majority class (nonintubated patients) and the minority class (intubated patients). We performed random undersampling [28] on the training data set to balance the majority class until both classes were equally balanced.

#### Transfer Learning Approach

The segmented CXRs were then fed into a pretrained DenseNet-201 [29] model. Multimedia Appendix 1 shows the architecture of the DenseNet-201 model. The DenseNet model was pretrained on RGB images (3 input channels) from ImageNet data set and on 1000 classes. Model input and output dimensions were changed to fit a grayscale image binary classification task. We also modified the architecture by adding a linear convolutional layer with a rectified linear unit activation function and dropout and used the LogSoftmax function to obtain the final probability output.

The model prespecifications were as follows: Adam optimizer, the loss function was binary cross-entropy, and the epoch size was 50. The framework used was PyTorch (version 1.01). Both segmentation and classification model training were performed using PyTorch libraries in Python [30] and trained with graphic processing unit clusters on Amazon Web Services in a secured network.

Then, using transfer learning [31], our binary classifier was trained using 10-fold cross-validation and a grid-search algorithm to tune hyperparameters (learning rate, number of hidden units, dropout, batch size) based on the area under the receiver operating characteristic curve (AUROC). Multimedia Appendix 2 shows the search ranges and the optimal hyperparameters.

#### Predictors in the Image Classifier

Convolutional neural network models lack decomposability into intuitive and understandable components, making them hard to interpret. To interpret our image classifier, we used the gradient-weighted class activation mapping method [32]. This technique provides us with a way to look into what particular parts of the image influenced the whole model’s decision for a specifically assigned label. It uses the gradients of our target label (intubation) flowing into the final convolutional layer to produce a coarse localization map, highlighting the important regions in the image for predicting the label. We tested our method on our test images, but this tool is yet to be automated.

### Model Fusion Classification: Combining EMR and CXR Data

**Figure 4:**
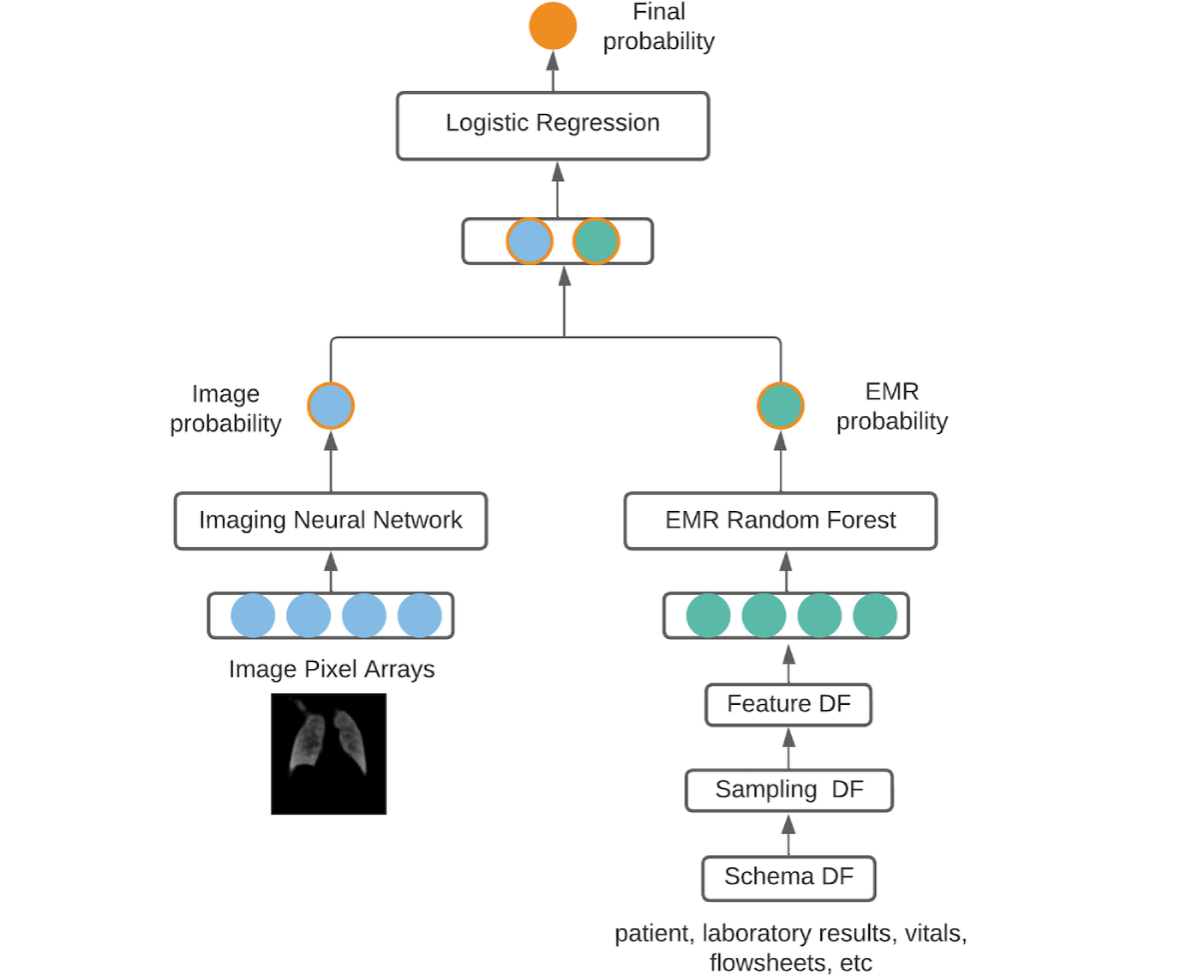
Fusion model architecture. The first 3 steps of the electronic medical record pipeline refer to output data frames. DF: data frame; EMR: electronic medical record.

When combining both data modalities—CXRs and EMR variables—different methods called fusion methods are possible [33]. The fusion model implemented here is a random forest [34] that has, as a feature vector, a concatenation of longitudinal features from the EMR (patient demographics, laboratory results, vitals, flowsheets) and the output probability from the image classifier. The final feature vector is described in Figure 4.

### Sampling Strategy for EMR Features

**Figure 5:**
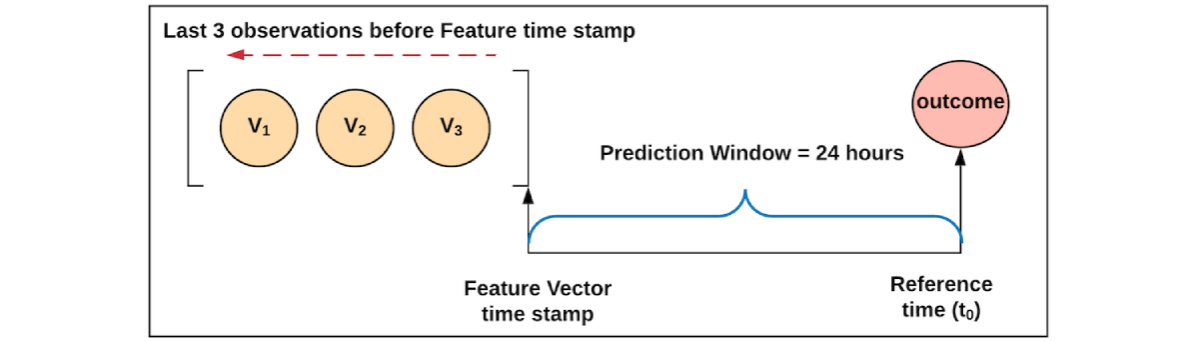
Sampling strategy flowchart for the electronic medical record variables.

Given the crisis nature of the pandemic, clinicians caring for this cohort collected data such as vital signs, laboratory results, electrocardiograms, and nursing assessments, based on clinical judgment and resource availability rather than standard protocols during the early phase of the crisis. Thus, to create longitudinal (time-series) data for each observational variable, we included the 3 most recent assessments available before the prediction time (Figure 5). Missing values for each variable were imputed using the median value across the cohort [35]. When less than 3 assessments are available for a particular variable, the available values are placed in the most recent time slots, and the oldest time-slot value is imputed with the cross-cohort median for that variable.

### EMR Feature Selection

From a total of 56 routinely collected EMR variables from the hospital, an optimal set of 41 variables was selected for the development of the predictive models (Multimedia Appendix 3). The variables initially removed included those with 90% or higher missing values and highly correlated variables [36] (above 0.7). We then performed recursive feature elimination [37]. In this approach, a single feature is removed at each step, and the model is evaluated on the test set. The quality of the fit to the data is measured using AUROC. Variables whose removal does not significantly alter the AUROC are eliminated from the feature set.

### Model Fusion Strategy

A random forest model was developed and optimized in Scala/Spark with the MLlib library [38] by using the training and test sets. It was trained using 10-fold cross-validation and a grid search algorithm to tune hyperparameters based on the AUROC on the test set to have robust evaluation.

### Model Testing and Statistical Methods

For each of the developed models, performance was evaluated on the test set and on the holdout set (which was not used for model development), and the model-derived class probabilities were used to predict intubation within 24 hours with a default threshold of 0.5. Predictions less than the threshold were categorized as negative. Sensitivity, specificity, accuracy, positive predictive value, negative predictive value, *F*_1_-score, AUROC, and area under the precision recall curve (AUPRC), along with bootstrap 95% CIs, were estimated for evaluating the screening tool’s performance. Group comparisons were performed using a 2-sided Student *t* test or Kruskal-Wallis for continuous variables as appropriate and chi-square test for categorical variables. All analyses were performed using SciPy in Python.

## Results

### Study Population and Outcomes

A total of 2481 COVID-19–positive patients were included in the overall study cohort. This cohort included a higher proportion of men (1390/2481, 56%), and the median age was 62.2 years. The median duration of hospital stay was 4.9 days and ranged from 1 to 72 days. The overall rate of intubation was 9.6% (239/2481) in the whole study cohort. Table 1 shows the clinical characteristics and descriptive statistics of the cohort. Intubated patients were significantly older and more likely to be male and diabetic than the nonintubated patients.

**Table 1:** Patient cohort and characteristics and statistical comparisons between intubated and nonintubated patient groups.

Characteristics		Overall (N=2481)	Intubated (n=239)	Nonintubated (n=2242)	*P* value
**Age (years)**					<.001
	Mean (SD)	60.4 (17.7)	64.9 (12.4)	59.9 (18.1)	
	Median (min-max)	62.2 (18-120)	65.5 (20-94)	62.0 (18-120)	
**Gender, mean (SD)**					.03
	Male	1390 (56.1)	135 (64)	1237 (55.2)	
	Female	1089 (43.9)	86 (36)	1003 (44.7)	
	Other	2 (0.08)	0 (0)	2 (0.1)	
**Race and ethnicity, mean (SD)**					<.001
	White	819 (32.9)	72 (30.1)	746 (33.3)	
	African American	456 (18.4)	24 (10)	433 (19.3)	
	Hispanic	600 (24.2)	65 (27.2)	536 (23.9)	
	Asian	129 (5.2)	13 (5.4)	116 (5.2)	
	Other	358 (14.4)	50 (20.9)	308 (13.7)	
	Unspecified	119 (4.8)	15 (6.3)	103 (4.6)	
**BMI**					.03
	Mean (SD)	29.4 (7.3)	30.5 (8.2)	29.3 (7.2)	
	Median (min-max)	28.3 (12.5-69.3)	28.7 (12.4-60.5)	28.3 (12.5-69.3)	
**Smoking history, mean (SD)**					.73
	Current smoker	24 (0.9)	1 (0.4)	23 (1)	
	Past smoker	558 (22.5)	48 (20.1)	510 (22.7)	
	Never smoked	78 (3.1)	9 (3.8)	69 (3.1)	
	Missing	1821 (73.4)	181 (75.7)	1640 (73.2)	
**Hypertension, mean (SD)**					.07
	Yes	1289 (51.9)	130 (54.4)	1159 (51.7)	
	No	1013 (40.8)	79 (33)	934 (41.7)	
	Missing	179 (7.2)	30 (12.6)	149 (6.6)	
**Diabetes, mean (SD)**					<.001
	Yes	854 (34.4)	112 (46.9)	742 (33.1)	
	No	1448 (58.4)	97 (40.5)	1351 (60.3)	
	Missing	179 (7.2)	30 (12.6)	149 (6.6)	
**Chronic obstructive pulmonary disease, mean (SD)**					.41
	Yes	399 (16.1)	41 (17.1)	358 (16)	
	No	1903 (76.7)	168 (70.3)	1735 (77.4)	
	Missing	179 (7.2)	30 (12.6)	149 (6.6)	
**Obesity, mean (SD)**					<.001
	Yes	445 (17.9)	64 (26.8)	381 (17)	
	No	1857 (74.9)	145 (60.7)	1712 (76.4)	
	Missing	179 (7.2)	30 (12.5)	149 (6.6)	
**Length of stay (days)**					.14
	Mean (SD)	6.6 (6.2)	7.2 (8.2)	6.5 (6.1)	
	Median (min-max)	4.9 (1-72)	4.7 (1-72)	4.9 (1-48)	
**Intensive care unit care received, mean (SD)**					<.001
	Yes	470 (18.9)	239 (100)	231 (10.3)	
	No	2011 (81.1)	0 (0)	2011 (89.7)	

### Predictors in the Final Fusion Model

**Figure 6:**
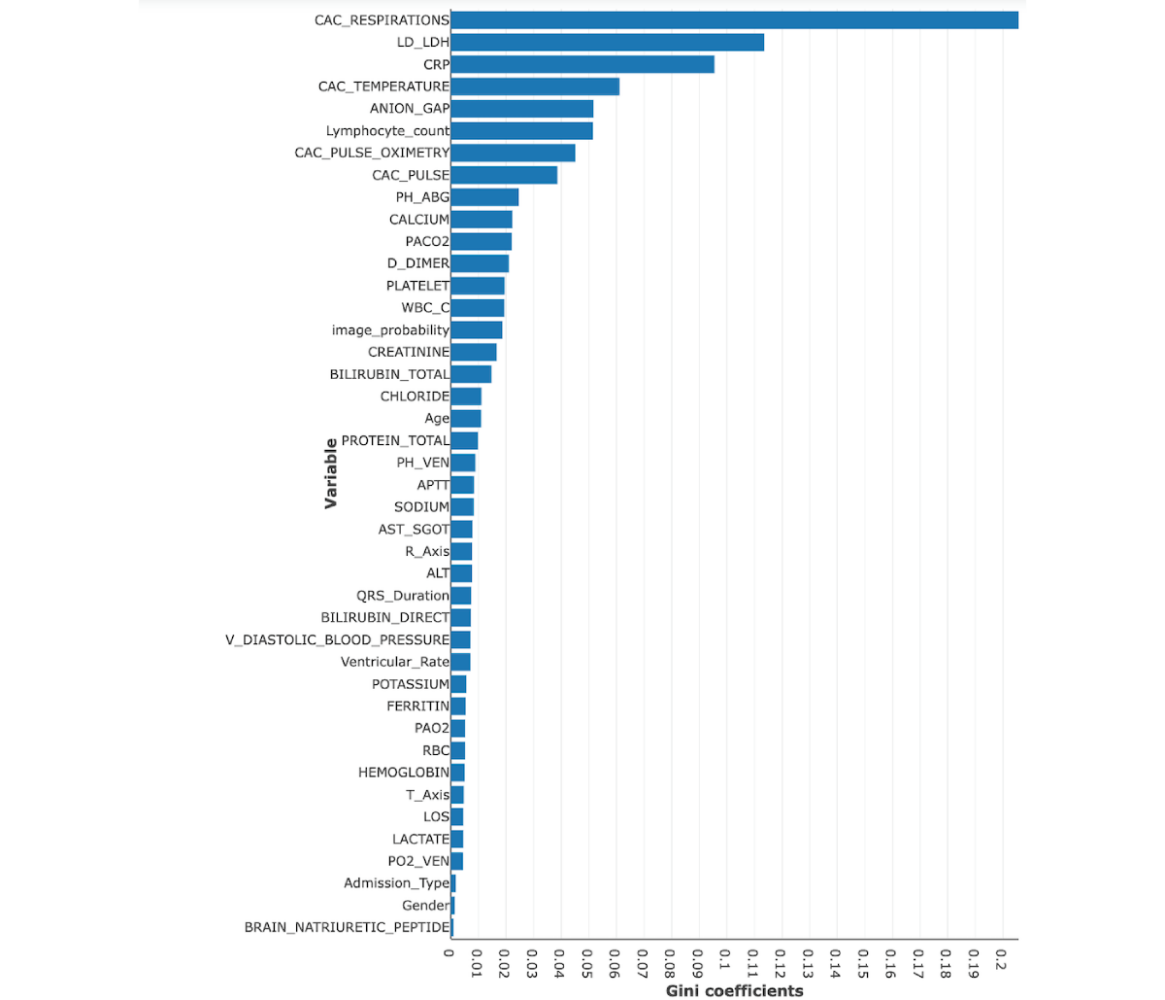
Gini coefficients of the joint fusion random forest model variables. Refer to Multimedia Appendix 3 for the definitions of the variables.

**Figure 7:**
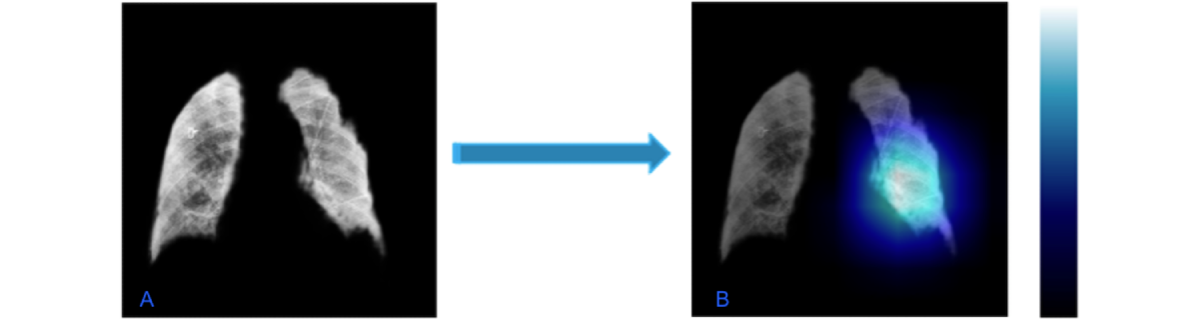
(A) Example of a segmented lung from the last chest radiograph of an intubated patient. (B) The most important features (pixels) predicting intubation are highlighted in the class activation map calculated by gradient-weighted class activation mapping projected on the image.

Hyperparameters used in the final random forest model are shown in Multimedia Appendix 2. Figure 6 summarizes the top predictive variables ordered by the Gini coefficient (the definitions of the variables in this figure are shown in Multimedia Appendix 3). Our model identified a series of features related to progressive respiratory failure (respiratory rate, oxygen saturation), markers of systemic inflammation (C-reactive protein, white blood cell count, lactate dehydrogenase), hemodynamics (systolic and diastolic blood pressures), renal failure (blood urea nitrogen, anion gap, and serum creatinine), and immune dysregulation (lymphocyte count). Respiratory rate (the earliest recorded value of the latest 3 assessments) had the highest predictive value in the random forest model, and white blood cell count was the second highest. Variables included in the final model reflected the importance of temporal changes in vital signs, markers of acid-base equilibrium and systemic inflammation, and predictors of myocardial injury and renal function. Figure 7 shows the parts of the lungs that contributed to intubation risk prediction.

### Comparison of the Predictive Performance of the Models

**Figure 8:**
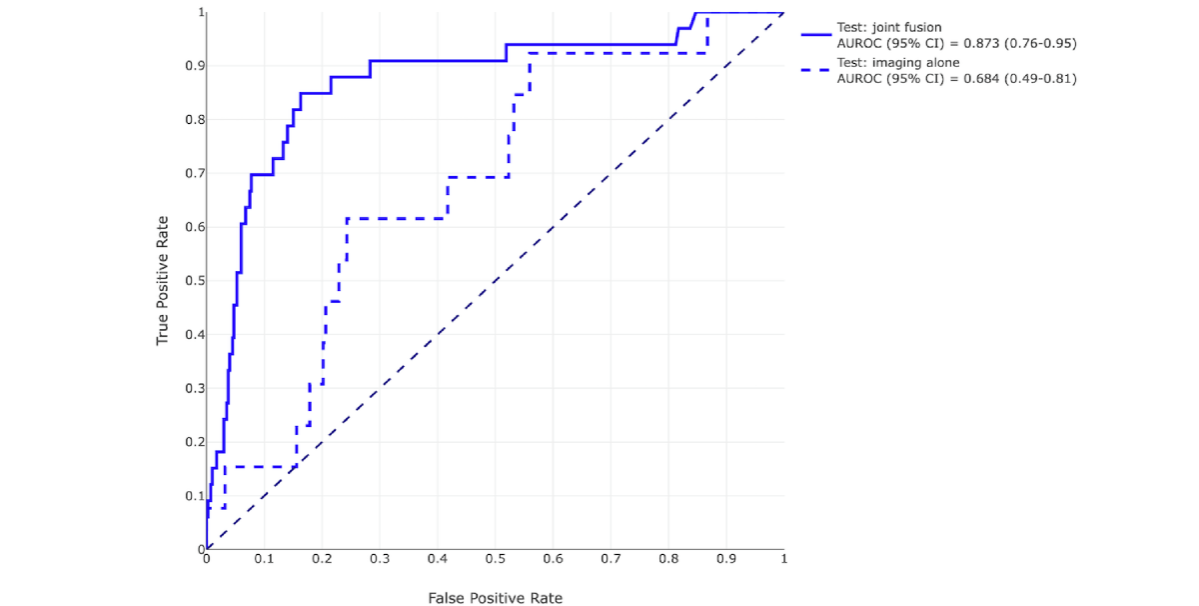
Receiver operating characteristic curves of the image classifier (blue dashed line) and of the joint fusion model (blue solid line) on the test set and their respective areas under the curve and 95% CIs. AUROC: area under the receiver operating characteristic curve.

**Figure 9:**
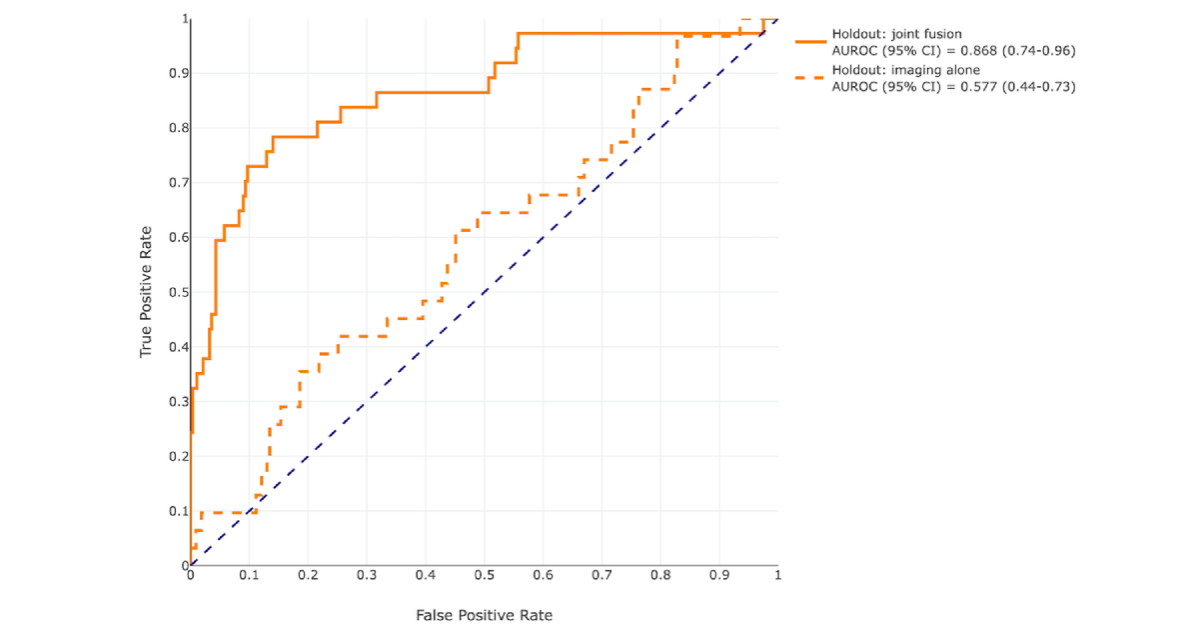
Receiver operating characteristic curves of the image classifier (orange dashed line) and of the joint fusion model (orange solid line) on the holdout set and their respective areas under the curve and 95% CIs. AUROC: area under the receiver operating characteristic curve.

At a prediction probability threshold of 0.5, the AUROC for the image classifier alone was 0.58 (95% CI 0.44-0.73) and the AUPRC was 0.21 (95% CI 0.08-0.38), with a positive predictive value of 14.8% (95% CI 7%-24%) on the holdout set. Table 2 shows all the performance metrics for all the models on the test set and the holdout set. Compared to the image classifier, the fusion model provided boosted performance results in the test set and the holdout set. By adding additional EMR features, the sensitivity doubled from 38.5% to 78.9%, specificity increased by nearly 10%, accuracy by 15%, positive predictive value by 104%, AUROC by 51%, *F*_1_-score by 112%, and the AUPRC by 140% in the holdout set. The AUROC graphs are shown in Figure 8 and Figure 9. The odds ratio for requiring mechanical ventilation within 48 hours of a positive prediction was 4.73 (95% CI 4.5-9.3) compared to a negative prediction and 11.2 (95% CI 10.4-12.0) for requiring mechanical ventilation at any time during admission in the holdout set.

**Table 2:** Predictive performance of both the image classifier and the joint fusion classifier on the test set and the holdout set. Positive and negative predictions were assigned using the prediction probability threshold of 0.5.

Data set, model		Sensitivity (95% CI)	Specificity (95% CI)	Accuracy (95% CI)	PPV^a^ (95% CI)	NPV^b^ (95% CI)	*F*_1_-score (95% CI)	AUROC^c^ (95% CI)	AUPRC^d^ (95% CI)	Unique patients (n)	Intubation rate
**Test**										432	0.076
	Imaging alone	0.5 (0.0-0.83)	0.776 (0.70-0.85)	0.757 (0.68-0.84)	0.103 (0.0-0.23)	0.965 (0.92-1.0)	0.160 (0.05-0.40)	0.684 (0.49-0.81)	0.124 (0.03-0.46)		
	Joint fusion	0.860 (0.67-1.0)	0.828 (0.78-0.89)	0.833 (0.78-0.88)	0.292 (0.16-0.43)	0.988 (0.96-1.0)	0.428 (0.27-0.58)	0.873 (0.76-0.95)	0.421 (0.19-0.64)		
**Holdout**										315	0.117
	Imaging alone	0.385 (0.15-0.64)	0.757 (0.68-0.84)	0.715 (0.63-0.79)	0.184 (0.07-0.32)	0.896 (0.82-0.95)	0.240 (0.09. 0.37)	0.577 (0.44-0.73)	0.206 (0.08-0.38)		
	Joint fusion	0.789 (0.59-0.96)	0.830 (0.76-0.89)	0.825 (0.76-0.88)	0.372 (0.22-0.54)	0.967 (0.93-0.99)	0.509 (0.34-0.67)	0.874 (0.80-0.94)	0.497 (0.32-0.65)		

^a^PPV: positive predictive value.

^b^NPV: negative predictive value.

^c^AUROC: area under the receiver operating characteristic curve.

^d^AUPRC: area under the precision recall curve.

## Discussion

In this study, we examined the utility of a deep learning image classifier based on routinely available CXR images along with clinical data to predict the need for IMV in patients with COVID-19. On the holdout set, the image classifier alone reached an AUROC of 0.58 and an AUPRC of 0.21; when the image probability was used in combination with structured EMR data in a random forest model, the fusion model reached an AUROC of 0.87 and an AUPRC of 0.50. Despite the relatively low AUPRC of the image classifier alone, it was still 15th in overall feature importance in the fusion model, outperforming some traditionally important clinical parameters such as creatinine levels, age, and venous blood pH. With optimization, a further increase in the feature importance of the image probabilities would be expected. The final fusion model had a negative predictive value of 97% and positive predictive value of 37% for the holdout set, which may provide significant clinical utility. This is supported by the fact that the odds ratio for intubation in patients with a positive prediction is greater than 11.

Several published reports have used deep learning of actual CXR images in combination with EMR data to predict the risk of intubation for patients admitted with COVID-19. Kwon et al [14], Aljouie et al [19], and Lee et al [39] used systematic manual scoring or manual labeling of CXR images to predict mechanical ventilation and deaths, achieving high performance; however, the utility of these approaches is limited, as it requires manual scoring by experts and cannot easily be rolled out to stressed health systems in an automated manner. Jiao et al [40] also used transfer learning on an ImageNet pretrained model to generate an image classifier used in fusion with EMR data to generate a classifier for intubation in patients with COVID-19 [40]. As in this study, the addition of EMR data boosted the image classifier performance, with the image classifier alone reaching an AUROC of 0.8, EMR alone reaching an AUROC of 0.82, and the fusion model an AUROC of 0.84. Although the addition of images only improved the AUROC of the EMR model from 0.82 to 0.84 in internal testing, on an external validation set, the addition of images improved AUROC from 0.73 to 0.79, which suggests that the images may be useful in guarding against overfitting. The differences between the image classifier and overall performance in the studies mentioned above and those in this study may be related to the higher event rate in their cohort, which diminished class imbalance (24% intubation rate in Jiao et al [40] vs 9.6% in this study) as well as potentially improved segmentation. Moreover, it suffers from manual review and hand editing of automated segmentation, which then limits clinical applicability versus using a fully automated imaging processing pipeline that this study offers.

Some studies utilized an end-to-end automated pipeline for processing radiography images and EMR data similar to that used in this study [17,22,24,41]; however, none make direct prediction of intubation and IMV in hospitalized patients. Chung et al [17] and Dayan et al [22] focused on the prediction of oxygen requirement in emergency department patients with limited data availability. Duanmu et al [41] focused on predicting the duration on IMV instead, but they are one of the very few using longitudinal data in their pipeline, suggesting that longitudinal data may bring more prognostic value than single-point data. O’Shea et al [24] had one of the highest performance end-to-end automated models, with an AUROC of 0.82 in predicting death or intubation within 7 days. However, those models are limited by the lack of image segmentation that ensure only pulmonary or thoracic features are considered in their models, use of a deep learning model to classify the degree of lung injury but not predict intubation itself, and use of a single point, that is, the first available value for each variable; therefore, they suffer from a lack of robustness that would not account for changes in the radiographs or in the patient’s clinical condition. The very long prediction window in O’Shea et al [24] (7 days vs 24 h in this study) is less amenable to clinical intervention.

The choice of a pretrained model may also be important. Kulkarni et al [18] used transfer learning using CheXNeXt, a DenseNet121 architecture model pretrained on a cohort of CXR images to identify lung pathologies as a base and reported an AUROC of 0.79 for their transfer learning model trained with only 510 images, suggesting that potentially fewer images are required when the model is pretrained on images closer to the appropriate subject matter [18].

The limitations of this study include a high-class imbalance of 9.6% (239/2481) intubation rate and a limited sample size of images. Another limitation was the changing practice pattern throughout the pandemic, as more was learned about the natural history of COVID-19, and practice patterns shifted to favor less frequent use of IMV [42]. Although there were fears of ventilator shortage or rationing of ventilators early in the pandemic; fortunately, there was no such shortage in the Mount Sinai Health System. Finally, the prediction time point for patients who were not intubated was selected to be 24 hours before discharge; this may potentially yield an optimistic performance benefit in this case, as patients are closer to recovery as opposed to deterioration and intubation. Further studies will demonstrate how much this affects performance. The strengths of this study include the use of a real-world clinical label of intubation that varied with practice patterns across the pandemic, use of a robust automated end-to-end pipeline that facilitated rapid deployment into the clinical setting, and fusion of image classifier and EMR classifier predictions in an interpretable manner such that the features most relevant to the prediction can be easily communicated to providers.

As the reach of deep learning and utilization of medical images in artificial intelligence–based clinical decision support increases, methods must be developed to combine these models with clinical data to optimize performance. Here, we demonstrate that, when linked with EMR data, an automated deep learning image classifier improved performance in identifying hospitalized patients with severe COVID-19 at risk for intubation. The image probability ranks highly among traditional clinical features in the relative importance of predictors. Further work is necessary to optimize the image classifier to yield higher performance and perform prospective and external validation. Ultimately, we seek methods that seamlessly integrate CXRs and other medical imaging with structured EMR data that enable real-time and highly accurate artificial intelligence clinical decision support systems.

## Appendix

Multimedia Appendix 1 Original DenseNet-201 architecture and modified architecture implemented in the intubation risk classification use case.

Multimedia Appendix 2 Hyperparameters used in the final imaging model.

Multimedia Appendix 3 Variables included in the final fusion model and their respective data source.

## Abbreviations

AUPRC: area under the precision recall curve
AUROC: area under the receiver operating characteristic curve
CXR: chest radiograph
DICOM: Digital Imaging and Communications in Medicine
EMR: electronic medical record
IMV: invasive mechanical ventilation
TRIPOD: Transparent Reporting of a Multivariable Prediction Model for Individual Prognosis Or Diagnosis

## Data Availability

Raw data underlying this study were generated by the Mount Sinai Health System. Derived data supporting the findings of this study are available from the corresponding author (Nguyen KAN) upon request.
