# Supplementary figures and images for: A Hybrid Decision Tree and Deep Learning Approach Combining Medical Imaging and Electronic Medical Records to Predict Intubation Among Hospitalized Patients With COVID-19: Algorithm Development and Validation
Source: JMIR Form Res. 2023 Oct 26;7:e46905. doi: 10.2196/46905 (PMC10636624; doi:10.2196/46905)

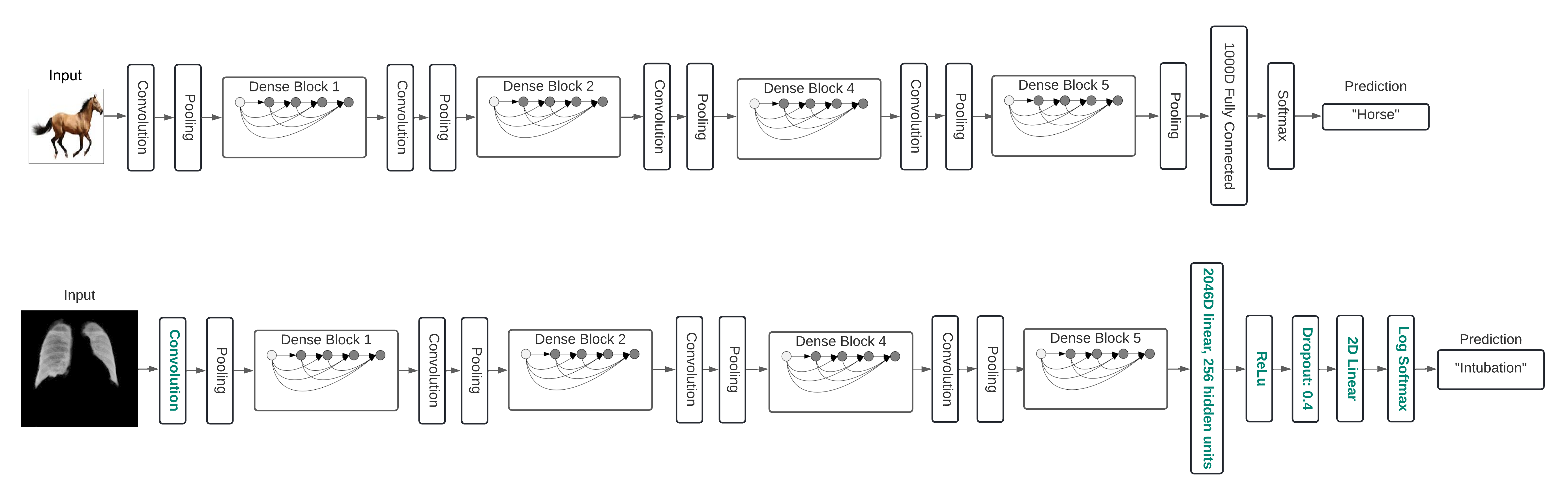

Supplement: Multimedia Appendix 1 [file formative_v7i1e46905_app1.png]
